# Laboratory and semifield data indicate that vector *Culicoides* spp. in Florida are susceptible to permethrin

**DOI:** 10.1093/jme/tjaf077

**Published:** 2025-06-26

**Authors:** Vilma M Cooper, Eva A Buckner, Juan M Campos-Krauer, Samantha M Wisely, Nathan Burkett-Cadena

**Affiliations:** Florida Medical Entomology Laboratory, Department of Entomology and Nematology, Institute of Food and Agricultural Sciences, University of Florida, Vero Beach, FL, USA; Florida Medical Entomology Laboratory, Department of Entomology and Nematology, Institute of Food and Agricultural Sciences, University of Florida, Vero Beach, FL, USA; Department of Large Animal Clinical Sciences, University of Florida, Gainesville, FL, USA; Department of Wildlife Ecology and Conservation, University of Florida, Gainesville, FL, USA; Florida Medical Entomology Laboratory, Department of Entomology and Nematology, Institute of Food and Agricultural Sciences, University of Florida, Vero Beach, FL, USA

**Keywords:** vector control, pyrethroids, insecticide resistance, *Culicoides*, permethrin

## Abstract

The genus *Culicoides* includes numerous species that are biting nuisances and vectors of pathogens affecting humans, livestock, and wildlife. For instance, *Culicoides paraensis* is the primary vector of Oropouche virus to humans, while other species, such as *Culicoides sonorensis*, *Culicoides insignis*, and *Culicoides venustus* transmit viruses causing hemorrhagic disease in ruminants, particularly bovids and cervids. Current control practices for biting midges rely heavily on chemical insecticides, yet their efficacy and potential for insecticide resistance in biting midges remain poorly documented. We conducted laboratory and semifield experiments to evaluate permethrin susceptibility in wild biting midges collected on 5 Florida deer farms, including known and suspected hemorrhagic diseasevector species, using lab-reared mosquitoes (*Aedes aegypti*) as a susceptible reference. The *Culicoides* species tested included: *C. insignis* (*n* = 944), *C. stellifer* (*n* = 269), *C. debilipalpis* (*n* = 68), *C. venustus* (*n* = 16), *C. edeni* (*n* = 5), *C. furens* (*n* = 5). Centers for Disease Control and Prevention bottle bioassays using 10.75 µg permethrin per bottle resulted in 100% mortality within 30 min across all *Culicoides* spp. Similarly, semifield cage trials with ultra-low volume permethrin-based space sprays applied at maximum label rate caused 100% mortality of all *Culicoides* spp. and susceptible *Ae. aegypti* within 1 h after exposure. Our findings confirm the effectiveness of permethrin for controlling biting midges and suggest that significant resistance to permethrin has not developed in the *Culicoides* populations tested. This study provides a foundation for optimizing vector control strategies against biting midges by demonstrating their susceptibility to permethrin and emphasizing the need for informed, evidence-based practices.

## Introduction

Vector control programs worldwide predominantly rely on chemical insecticides to manage pathogen vectors (Van Den Berg et al. 2021). Pyrethrins and pyrethroids are widely used for controlling blood-feeding dipterans, such as mosquitoes and biting midges (Walters et al. 2009) and permethrin is the most commonly used pyrethroid because of its high insecticidal potency and rapid action (Wang et al. 2016). However, widespread pyrethroid use has raised concerns about the development of insecticide resistance in mosquitoes (Zulfa et al. 2022, Uemura et al. 2024).

Biting midges (Diptera: Ceratopogonidae: *Culicoides*), commonly known as no-see-ums, are vectors of arboviruses that impact both veterinary and public health (Mullen and Murphree 2019). *Culicoides paraensis* Goeldi, for example, is the primary vector of Oropouche virus (OROV), which causes febrile illness in humans (Mohapatra et al. 2024). In the United States, biting midges transmit bluetongue virus (BTV) and epizootic hemorrhagic disease virus (EHDV), which cause hemorrhagic disease (HD) in ruminants. While *Culicoides sonorensis* Wirth & Jones (EHDV and BTV) and *Culicoides insignis* Lutz (BTV) are the only 2 confirmed vectors of HD viruses in North America (Foster et al. 1977, Jones et al. 1977, Tabachnick 2004), other species, including *Culicoides debilipalpis* Lutz, *Culicoides obsoletus* Meigen, *Culicoides spinosus* Root & Hoffman, *Culicoides stellifer* (Coquillett), *C. paraensis* and *Culicoides venustus* Hoffman have been implicated as potential vectors (Mullen et al. 1985, Smith and Stallknecht 1996, McGregor et al. 2019, 2021).

In Florida, the management of biting midges is particularly relevant on deer farms, where high deer densities elevate HD transmission potential (McGregor et al. 2019). Permethrin-based insecticides are the primary control tool against biting midges, with 86% of Florida deer farmers reporting regular use (Harmon et al. 2020). However, frequent applications, sometimes daily during peak HD transmission season, may not align with insecticide label recommendations which are designed to minimize environmental impact and delay resistance (Harmon et al. 2020, Cooper et al. 2025).

Insecticide resistance and efficacy monitoring are essential for maintaining the effectiveness of vector control programs (McAllister and Scott 2020). Laboratory bioassays, such as the World Health Organization (WHO) tube assay and the Centers for Disease Control and Prevention (CDC) bottle bioassay, are commonly used to assess susceptibility of adult vectors to technical grade active ingredients (Brogdon and Chan 2010), and semifield cage trials provide data on the effectiveness of formulated insecticide space spray products against vectors under field conditions (WHO 2009, McAllister and Scott 2020). Although these bioassays were developed for mosquitoes, they have been adapted for use in other vector groups. However, their application to biting midges has been limited, and standardized methods have only recently been developed (Cooper et al. 2025).

Few studies have assessed biting midge susceptibility to insecticides in the United States. Early work on the coastal pest species *Culicoides furens* (Poey) in Florida evaluated the efficacy of space sprays containing organophosphates (malathion, naled) and the pyrethroid resmethrin, all applied at twice the label dosage (Linley et al. 1987, 1988, Linley and Jordan 1992). These studies found naled to be the most effective, achieving 90% mortality at distances up to 106 m, compared to 36 m for malathion, and 25 m for resmethrin. More recently, Cooper et al. (2025) demonstrated that permethrin resulted in 100% mortality of *C. furens* from Vero Beach, FL during semifield cage trials and CDC bottle bioassays.

As permethrin remains the primary adulticide (insecticide used for adult vector management) for mosquito control agencies (Lloyd et al. 2018), and deer farmers in Florida (Harmon et al. 2020, Cooper et al. 2025), evaluating its efficacy against biting midges is critical to generate baseline information and allow insecticide resistance monitoring in the future. This study assesses the susceptibility of field-collected biting midges to permethrin using recently developed laboratory and semifield methods (Cooper et al. 2025). These findings aim to inform sustainable and effective vector management practices to mitigate the impact of biting midges as vectors and pests.

## Materials and Methods

Laboratory and semifield experiments were conducted at 5 study sites in Florida to evaluate permethrin susceptibility of field-collected (wild) *Culicoides* spp., including putative and confirmed HD vector species. Experiments consisted of CDC bottle bioassays and semifield cage trials, following the methodology described in Cooper et al. (2025).

### Insects

Field-collected *Culicoides* biting midges were used for all experiments. Using wild biting midges has been somewhat normalized due to challenges in their colonization (McGregor et al. 2021). Biting midges were trapped at 5 deer farms using CDC miniature light traps (BioQuip, Rancho Dominguez, CA, USA) connected to live midge collection chambers as described by Erram and Burkett-Cadena (2018). Traps were baited with an incandescent light bulb and ~1 kg of dry ice contained in an insulated 1.89 l beverage container (Igloo Products Corp., Katy, TX, USA). Biting midges were brought to a temporary laboratory station at or near the farm the morning after collection, and allowed to acclimate to indoor conditions until the time of the bottle bioassay or cage trial (6 to 8 h later). Biting midges were provided with cotton rounds soaked with a 10% sucrose solution as a carbohydrate source until the time of the experiment.

Laboratory-reared *Aedes aegypti* (Linnaeus) were used as the susceptible species of reference due to the lack of biting midge colonies. This laboratory-reared strain of *Ae. aegypti* originated in Orange County (Orlando, FL, 1952) and was obtained from colonies maintained at the USDA-ARS CMAVE. Egg papers were placed in plastic trays with 2 liter of tap water at a density of 200 to 300 eggs per tray. Larvae were fed a 1:1 mixture of lactalbumin and yeast ad libitum. Pupae were transferred to water-filled plastic cups in 30.5 × 30.5 × 30.5 Bug Dorm adult rearing cages (BioQuip, Rancho Dominguez, CA, USA). Emerging adults were provided with cotton rounds soaked with 10% sucrose solution as a carbohydrate source.

### Study Sites

**Fig. 1.**
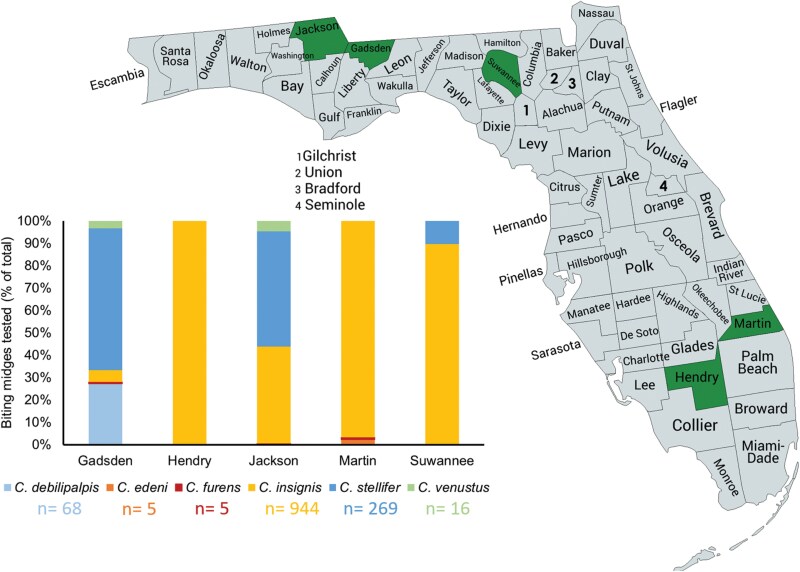
Map of study sites and species used for experiments. Counties highlighted represent the location of study sites (deer farms) where CDC bottle bioassays and semifield cage trials were performed to evaluate permethrin susceptibility in Culicoides biting midges. *N* represents the total number of biting midges used for one bottle bioassay (5 bottles), and one field cage trials (7 cages) per farm

The 5 deer farms used for experiments were selected from a list of Florida deer farms that have previously permitted sample collection (insect and deer necropsy) by the University of Florida, Institute for Food and Agricultural Science, Cervidae Health Research Initiative (Sloyer et al. 2019, Cottingham et al. 2021). These farms are located in Gadsden, Hendry, Jackson, Martin, and Suwannee County in Florida (Fig. 1). All of these deer farms regularly perform applications of permethrin-based insecticides using vehicle-mounted ULV adulticiding machines as the main strategy for the control of adult biting midges and have reported HD cases and HD vector species on their properties in recent years. For instance, preliminary studies at the farms in Martin County and Hendry County found high density of the BTV vector, *C. insignis* (Cooper, unpublished data). This species has also been found on the farm in Suwannee County, along with high relative abundance of *C. stellifer,* a putative vector of EHDV (Sloyer et al. 2019). The farm in Gadsden County harbors a large diversity of biting midges, including the putative vectors of EHDV, *C. venustus* and *C. stellifer*. In fact, this farm is one of the locations where data to incriminate these 2 species as vectors of EHDV were collected (McGregor et al. 2019). In addition, preliminary trapping in the fall of 2022 in the Jackson County farm confirmed the presence of *C. stellifer*, *C. venustus*, and *C. insignis* (Cooper, unpublished data).

### CDC Bottle Bioassay

At each deer farm, a CDC bottle bioassay was performed, consisting of 5 250 ml clear glass Wheaton bottles with lids (DWK Life Sciences Inc., Millville, NJ), 4 of which were coated with 1.0 ml of a permethrin–acetone solution (10.75 µg permethrin/bottle) and 1 coated with 1.0 ml of acetone only (Cooper et al. 2025). Bottles were coated by swirling the solution inside the bottle and then placed on a hot dog roller with heat turned off until all visible signs of the liquid were gone (Brogdon and Chan 2010). After coating, the caps were removed and bottles were left to dry in a dark room for 24 h before the experiment (Cooper et al. 2025).

Bioassays were performed at a laboratory station at or near the farms, where the temperature was constant (25 to 30 °C). Biting midges (~20 adult females) and *Ae. aegypti* (~20 adult females and males) were aspirated by mouth into each bottle. The diagnostic time of 30 min was used (Cooper et al. 2025). Mortality was recorded every 5 min for the first 15 min, and every 15 min until 2 h postexposure (McAllister and Scott 2020). After 2 h, insects were transferred into 0.2 liter paper cups with lids and no-see-um netting (Seattle Fabrics, Inc., Seattle, WA) to prevent insects from escaping. Insects were provided with a cotton round with a 10% sucrose solution and mortality was then assessed at 24 h postexposure (Parker 2020).

### Semifield Cage Trials

One semifield cage trial using live insects was conducted per farm during a routine permethrin application performed by a farmer using a vehicle-mounted ULV adulticiding machine. Bioassay cages consisted of cardboard rings (15.2 cm diameter, 2.5 cm height) covered with no-see-um netting (Seattle Fabrics, Inc., Seattle, WA). Biting midges (~20 females) and *Ae. aegypti* (~20 adult females and males) were aspirated by mouth into the same cage. Cages were hung from shepherd hooks spaced every 5 m located 20 m downwind (*n* = 5 treated cages) and 20 m upwind (*n* = 2 untreated control) from the spray vehicle path. The adulticide spraying equipment consisted of a ULV aerosol generator mounted on a golf cart (Hendry, Jackson, and Martin County), a truck (Gadsden County), or a tractor (Suwanee County). All applications took place in the evening, between 6 PM and 7 PM EST. Information on the product applied, the speed of the application vehicle, and wind speed was recorded (Table 1).

**Table 1.** Summary of semifield cage trials to evaluate the efficacy of ULV permethrin space spray applications against field-collected *Culicoides* biting midges on five deer farms in Florida, 2023 to 2024. All farms applied the permethrin product undiluted

County and date	Product	Active ingredients (AI)	Speed of application vehicle (mph)	Wind speed (mph)	Application rate (g AI/ha)
Gadsden 4 October 2023	PermaSease 4-4	Permethrin (4.5%), PBO^a^ (4.6%)	5	5	7.85
Hendry 23 August 2023	Pursuit 4-4	Permethrin (4.6%), PBO (4.6%)	5	5	7.85
Jackson 17 June 2023	Perm-X UL 4-4	Permethrin (4%), PBO (4%)	5	4	7.85
Martin 3 August 2023	PermaSease 4-4	Permethrin (4.5%), PBO (4.6%)	5	5	7.85
Suwannee 19 August 2024	Permanone 30-30	Permethrin (30%), PBO (30%)	5	4	7.85

^a^PBO: piperonyl butoxide.

Mortality in bioassay cages was assessed at 10 min, 1, 12, and 24 h postexposure, based on knockdown (inability to stand on their legs or have coordinated flight) ( McAllister and Scott 2020). Ten minutes postspray, bioassay cages were retrieved from the shepherd hooks and stored in plastic totes covered with a damp towel. Untreated control bioassay cages were stored in a separate container from treated bioassay cages, and all cages were provided with a cotton round with a 10% sucrose solution as carbohydrate source. Final mortality was assessed at 24 h, after which cages were placed in coolers containing dry ice to kill any remaining live insects. Insects were counted and biting midges were identified to species using dichotomous keys (Blanton and Wirth 1979, Blosser et al. 2024).

### Statistical Analysis

CDC bottle bioassay data were analyzed with a Cox proportional hazards model using the “coxph” function from the survival R package (Burgess et al. 2022). A separate model and survival plot was created for each farm. Treatment (permethrin or untreated control) was included as a predictor, and replicates (bottles) were clustered.

Semifield cage trial data were analyzed using descriptive and inferential statistics. Average mortality was calculated for biting midges and *Ae. aegypti* at 10 min, 1, 12, and 24 h postapplication and plotted for each farm. Due to variability in field-collected biting midges, species-specific analyses were not performed. Species diversity used for experiments was summarized and visualized for each farm.

## Results

CDC bottle bioassays and field cage trials were conducted during HD season in Florida (Fall) in 2023, except for the Suwannee County farm, where experiments took place in late summer of 2024 due to damages to the farm caused by hurricane Ian in 2022. The biting midge species used in the bioassays varied across farms but included at least one of the following species known or suspected to transmit HD viruses: *C. debilipalpis*, *C. insignis*, *C. stellifer,* and *C. venustus* (Fig. 1).

### CDC Bottle Bioassays

**Fig. 2.**
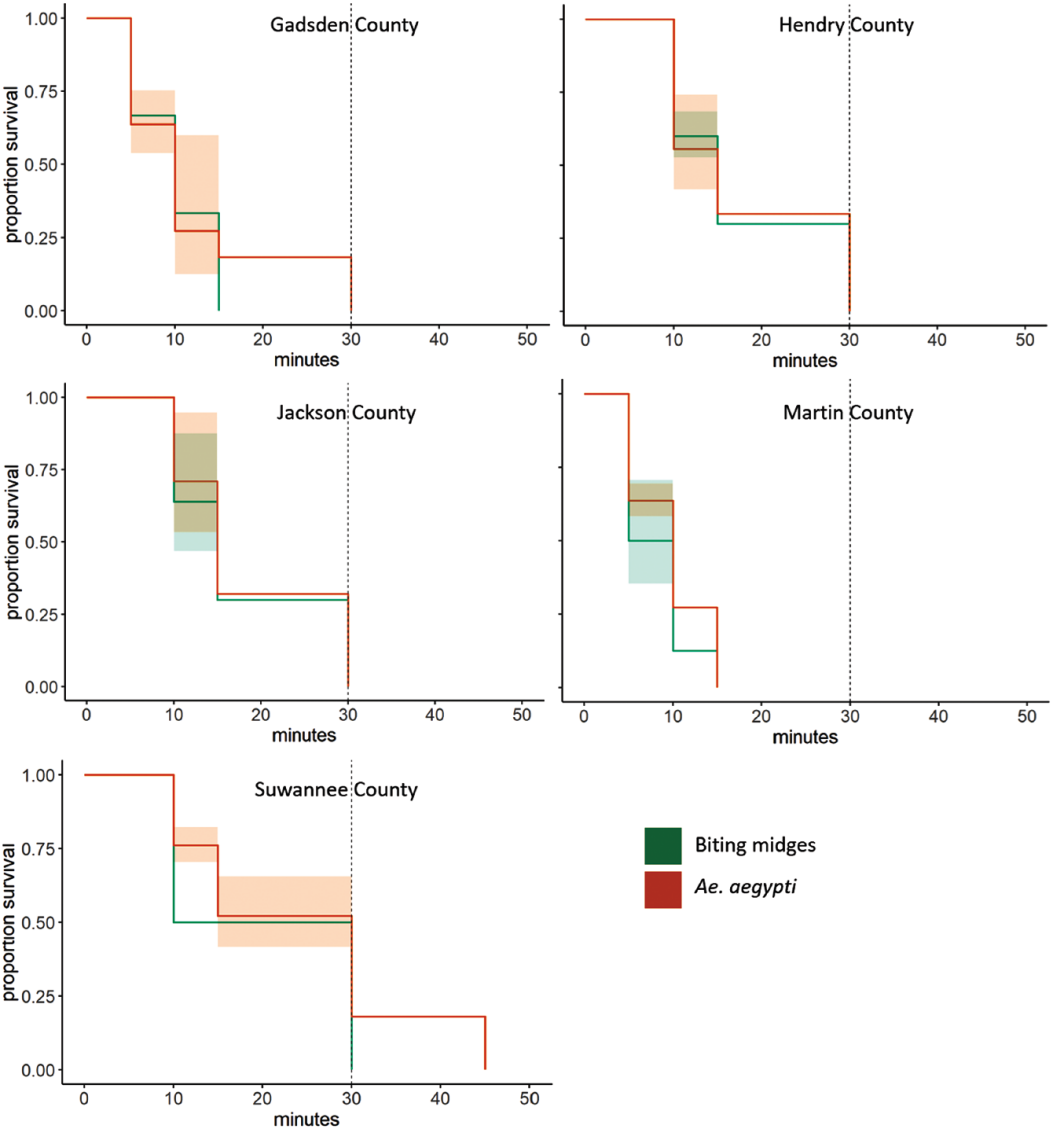
Survival curves and 95% confidence intervals (shaded area) for Culicoides biting midges from 5 Florida deer farms and susceptible *Aedes aegypti* in CDC bottle bioassays performed with permethrin (10.75 µg permethrin/bottle).

At the diagnostic dose of 10.75 µg permethrin/bottle, complete mortality (100%) of biting midges occurred by the diagnostic time of 30 min within all bottle bioassays performed (Fig. 2). Specifically, mortality began within the first 5 min of exposure, reaching 100% by 15 min in the bottle bioassays performed in Gadsden and Martin County, and by 30 min in the other locations (Fig. 2). *Aedes aegypti* experienced 100% mortality within 2 h of exposure. In most bottle bioassays, 100% mortality in *Ae. aegypti* occurred rapidly, by 15 min in Martin County, 30 min in Gadsden and Hendry County, 30 min in Jackson County, and 45 min in Suwanee County (Fig. 2).

Cox proportional hazards analysis revealed no statistically significant difference in the hazard ratio (HR) between biting midges and susceptible mosquitoes for Gadsden County (HR = 0.73, *P* = 0.20), Hendry County (HR = 1.00, *P *= 0.98), and Jackson County (HR = 0.76, *P* = 0.4705). This suggested similar susceptibility profiles between the 2 groups. In Martin County, the HR indicated that biting midges had lower survival rates than mosquitoes (HR = 0.54, *P* = 0.0051). Similarly, mosquitoes had higher survival rates relative to biting midges in Suwannee County, (HR = 0.59, *P* = 0.0019). Survival analysis across all counties showed a highly significant difference in mortality between exposed and unexposed (control) individuals (HR ≈ 0, *P* < 0.0001), confirming the impact of permethrin exposure across both species.

### Semifield Cage Trials

Complete mortality (100%) was observed within 1 h after exposure to the ULV application for all field-collected biting midges and the susceptible *Ae. aegypti* across the 5 farms (Fig. S1), and no knockdown recovery was observed at 24 h postexposure. Little to no mortality of biting midges and *Ae. aegypti* occurred within 10 min postapplication. Untreated control cages showed no mortality of *Ae. aegypti* or biting midges on most farms, except for Martin County, where 60% of control biting midges were dead at 24 h postexposure (Fig. S1), likely due to stress during transportation. Because control mortality at 24 h exceeded acceptable thresholds for interpretation, we did not include the Martin County 24-h data in forming our conclusions. However, given that 100% mortality was already achieved by 12 h and no recovery was observed at other sites, we believe it is reasonable to assume that recovery was unlikely in Martin County as well.

## Discussion

This study demonstrated that 6 species of biting midges collected from 5 Florida deer farms are susceptible to permethrin, as shown through CDC bottle bioassays assessing insecticide resistance and semifield cage trials evaluating the efficacy of ULV applications. These complementary approaches provide a more complete understanding of how permethrin may perform in both controlled and field conditions. These findings have significant implications for controlling biting midge nuisance species and improving livestock health, particularly on deer farms affected by biting midge-transmitted viruses such as EHDV and BTV.

Despite the widespread use of adulticides to control biting midges, the susceptibility of these insects to widely used insecticides remains poorly documented. Our results highlight the utility of CDC bottle bioassays for assessing insecticide resistance and semifield cage trials for evaluating adulticide efficacy against veterinary significant biting midge species. These methods could also be potentially extended to medically relevant biting midge species, such as *C. paraensis,* which has recently gained attention due to its role in the transmission of Oropouche virus to humans.

The CDC bottle bioassays demonstrated that the diagnostic dose of 10.75 µg permethrin per bottle (Cooper et al. 2025) achieved 100% mortality of *C. insignis*, *C. edeni*, *C. furens*, *C. stellifer*, *C. venustus*, and *C. debilipalpis* within 30 min of exposure. These results align with the only previous CDC bottle bioassay data, where *C. furens* from Vero Beach, FL, experienced 100% mortality with the same diagnostic dose and time (Cooper et al. 2025). While the Vero Beach site where Cooper et al. (2025) collected *C. furens* is characterized by extensive coastal hammock and mangrove swamps, biting midges may be exposed to ULV permethrin space spray applications performed by the local mosquito control district, potentially influencing their susceptibility. However, we believe that the findings of our current study support the use of 10.75 µg as the diagnostic dose and 30 min as the diagnostic time in bottle bioassays performed with permethrin against wild *Culicoides* spp. as suggested in Cooper et al. (2025). Furthermore, these results establish baseline information that can be used in the future to monitor deviations that may indicate resistance development.

The time to reach 100% mortality in the susceptible reference, *Ae. aegypti*, varied among farms, from 15 to 45 min. In another study, *Ae. aegypti* from this colony exposed to the same diagnostic dose experienced 100% mortality by 45 min (Cooper et al. 2025). We suspect that the rapid mortality (15 min) of *Ae. aegypti* in the Martin County bioassay may have been influenced by unusual temperature fluctuations experienced during travel between the farm and the laboratory station where bioassays were performed. Notably, biting midges from the same site also experienced one of the fastest knockdown among all locations tested. Given the parallel pattern in both species, it is reasonable to assume that the same environmental factors contributing to increased susceptibility in *Ae. aegypti* may have similarly affected the response of biting midges.

In semifield cage trials, some variability in mortality at 10 min was observed among farms (Fig. S1). For instance, no mortality of biting midges was observed at 10 min in Hendry and Martin County, while nearly 50% mortality occurred in Suwanee County (Fig. S1). These differences were likely due to the different synergized permethrin-based formulations and application rates (Table 1). Notably, all the formulated permethrin products used in cage trials contained piperonyl butoxide (PBO), a synergist that inhibits insect detoxification enzymes and enhances the efficacy of pyrethroids ( Schluep and Buckner 2021). The inclusion of PBO is especially important for mitigating the impact of emerging resistance in field populations, a strategy commonly used in mosquito control (Joseph et al. 2024). Although technical grade permethrin alone achieved 100% mortality in bottle bioassays, the addition of PBO in formulated products may extend the useful lifespan of permethrin by delaying resistance development.

While permethrin susceptibility data for *C. debilipalpis*, *C. edeni*, *C. insignis*, *C. stellifer*, and *C. venustus* were previously unavailable, our results indicate that permethrin-based space spray products are effective for control of these species. Previous insecticide trials in the United States focused on nuisance species like *C. furens* and reported suboptimal outcomes with nonpermethrin products (Linley et al. 1987, 1988, Linley and Jordan, 1992). However, a recent study demonstrated that ULV applications of a permethrin-based adulticide space spray (Permanone 30-30) applied at maximum label rate (0.007 lb, permethrin/acre) achieved 100% mortality of *C. furens* (Cooper et al. 2025). Our results support these findings, reinforcing the efficacy of permethrin space spray products for controlling biting midges. While our results indicate that ULV permethrin causes 100% mortality in the6 species tested, we cannot confirm whether there was a decline in the biting midge population in the farm. Future research should focus on the impact of permethrin-based ULV applications on biting midge density and virus transmission.

A key limitation of the CDC bottle bioassays and semifield cage trials involving mixed *Culicoides* species is the difficulty in interpreting results when complete morality does not occur. In this study, complete mortality by the diagnostic time simplified interpretation. However, if future assays achieve only partial mortality, it would be challenging to determine which species contributed to that outcome without conducting species-level identification at multiple time points, an approach that is logistically difficult within bottle or cage settings. Whenever possible, identifying individuals to species prior to experimentation would help mitigate this issue and improve the interpretability of results, particularly when monitoring for early signs of resistance. Alternatively, individual bottle bioassays using a single insect per bottle, as demonstrated by Balaska et al. (2024), may offer a valuable approach to distinguish species-specific responses in mixed populations.

Our findings demonstrate that ULV applications of a permethrin-based adulticide space sprays cause complete mortality in biting midge species of veterinary importance, including known and putative vectors of EHDV and BTV: *C. debilipalpis*, *C. insignis*, *C. stellifer*, and *C. venustus*. This information can benefit deer farmers, particularly in Florida, where HD accounts for nearly half of the reported mortalities in farmed deer (Cottingham et al. 2021). Permethrin space sprays against biting midges may also have broader applications beyond deer farming. For instance, *C. insignis* is not only the primary vector of BTV in the United States, Central and South America, but also contributes to economic losses by causing allergic dermatitis in livestock, particularly sheep (Corrêa et al. 2007). Permethrin space spray applications in these systems could enhance animal health and productivity, potentially making it a valuable component of integrated vector management programs.

Evaluating the efficacy of permethrin space sprays is essential for the management of biting midges, especially since this remains the main control strategy against biting midges in Florida (Harmon et al. 2020, Cooper et al. 2025). Effective control strategies for *Culicoides* spp. often combine insecticides, repellents, animal stabling, vaccination, and habitat elimination (Pfannenstiel et al. 2015, Harrup et al. 2016). However, specific measures targeting EHDV and BTV vectors in the United States remain underdeveloped, hindered by limited knowledge of *Culicoides* biology (Benelli et al. 2017, Erram and Burkett-Cadena 2018) and logistical constraints (Corrêa et al. 2007, Orange et al. 2021). Therefore, ensuring the continued efficacy of permethrin space sprays is essential for reducing costs and delaying the development of insecticide resistance, improving control outcomes while enhancing sustainability.

We did not detect resistance to permethrin among the tested biting midge species. However, we recommend integrating resistance monitoring into routine farm management to ensure long-term efficacy of permethrin space spray treatments. Integrating other methods, such as vaccinating deer against EHDV and eliminating breeding sites, could further improve control outcomes (Carpenter et al. 2008, Harrup et al. 2016, Wisely and Campos-Krauer 2020). Overall, a holistic integrated vector management approach is necessary for sustainably protecting deer from biting midges and the pathogens they transmit (Pfannenstiel et al. 2015). This study provides a foundational step toward achieving this goal, emphasizing the importance of informed vector control practices.

## Supplementary Material

tjaf077_suppl_Supplementary_Figure_S1
