# Supplementary figures and images for: Laboratory and semifield data indicate that vector Culicoides spp. in Florida are susceptible to permethrin
Source: J Med Entomol. 2025 Jun 26;62(5):1235–42. doi: 10.1093/jme/tjaf077 (PMC12507431; doi:10.1093/jme/tjaf077)

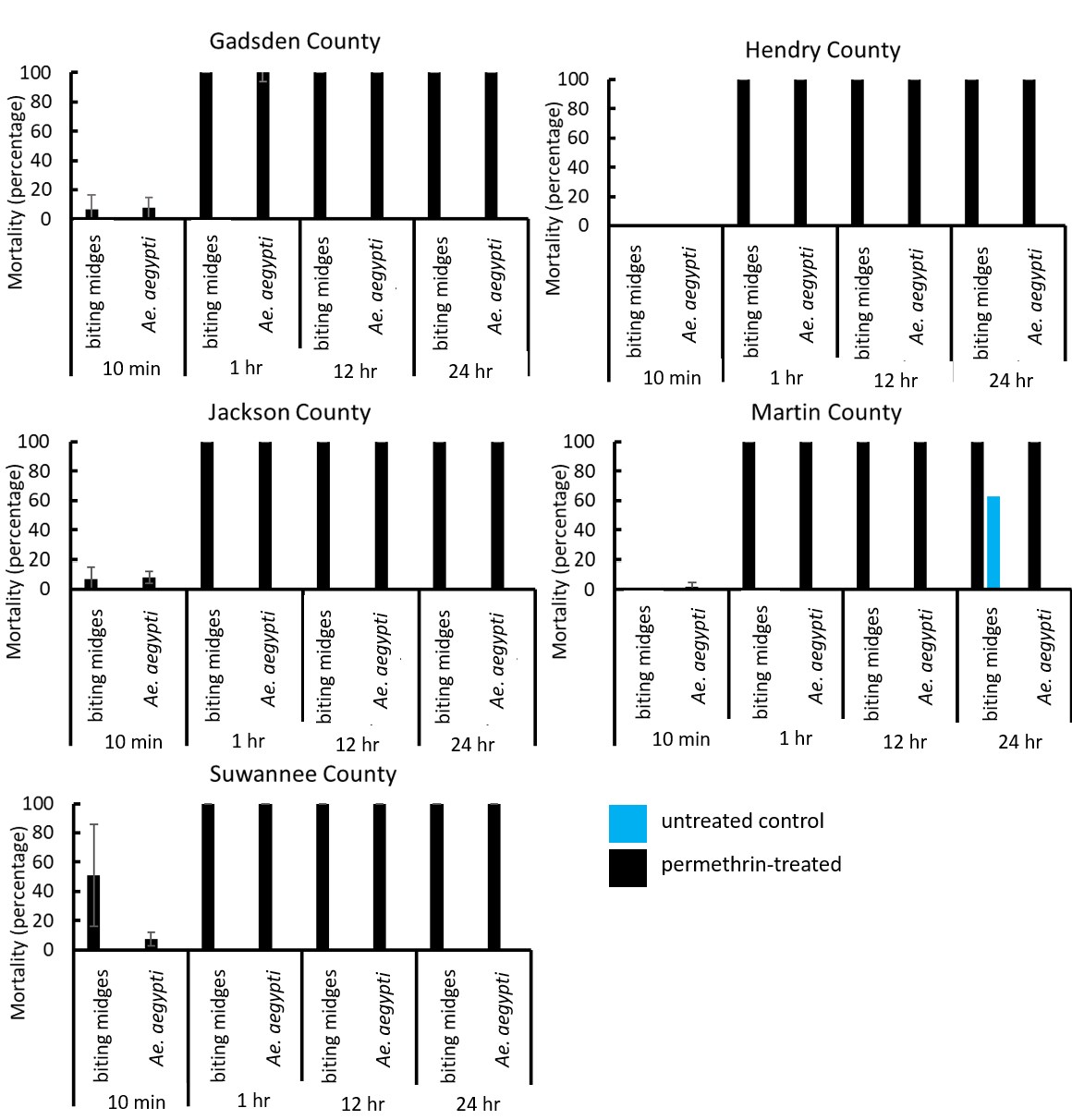

Supplement: tjaf077_suppl_Supplementary_Figure_S1 [file tjaf077_suppl_supplementary_figure_s1.jpeg]
